# Cannabis and Endometriosis: The Roles of the Gut Microbiota and the Endocannabinoid System

**DOI:** 10.3390/jcm12227071

**Published:** 2023-11-13

**Authors:** Toobah Farooqi, Deep Jyoti Bhuyan, Mitchell Low, Justin Sinclair, Mathew Leonardi, Mike Armour

**Affiliations:** 1 NICM Health Research Institute, Western Sydney University, Sydney 2751, Australia; t.farooqi2@westernsydney.edu.au (T.F.); d.bhuyan@westernsydney.edu.au (D.J.B.); mitchell.low@westernsydney.edu.au (M.L.); j.sinclair@westernsydney.edu.au (J.S.); 2 School of Science, Western Sydney University, Sydney 2751, Australia; 3 Department of Obstetrics and Gynecology, McMaster University, Hamilton, ON L8V 5C2, Canada; leonam@mcmaster.ca; 4 Robinson Research Institute, University of Adelaide, Adelaide 5006, Australia; 5 Translational Health Research Institute, Western Sydney University, Sydney 2751, Australia; 6 Medical Research Institute of New Zealand, P.O. Box 7902, Wellington 6242, New Zealand

**Keywords:** endometriosis, the endocannabinoid system, CB_1_, CB_2_, gut microbiota

## Abstract

Endometriosis, a chronic condition affecting around 10–14% of women, is challenging to manage, due to its complex pathogenesis and limited treatment options. Research has suggested a potential role of the gut microbiota and the endocannabinoid system in the development and progression of endometriosis. This narrative review aims to explore the role of, and any potential interactions between, the endocannabinoid system (ECS) and the gut microbiota in endometriosis. This review found that both the ECS and microbiota influence endometriosis, with the former regulating inflammation and pain perception and the latter influencing immune responses and hormonal balance. There is evidence that a dysregulation of the endocannabinoid system and the gut microbiota influence endometriosis symptoms and progression via changes in CB1 receptor expression and increased circulating levels of endocannabinoids. Microbial imbalances in the gut, such as increases in *Prevotella*, have been directly correlated to increased bloating, a common endometriosis symptom, while increases in *E. coli* have supported the bacterial contamination hypothesis as a potential pathway for endometriosis pathogenesis. These microbial imbalances have been correlated with increases in inflammatory markers such as TNF-α and IL-6, both often raised in those with endometriosis. Protective effects of the ECS on the gut were observed by increases in endocannabinoids, including 2-AG, resulting in decreased inflammation and improved gut permeability. Given these findings, both the ECS and the gut microbiota may be targets for therapeutic interventions for endometriosis; however, clinical studies are required to determine effectiveness.

## 1. Introduction

Endometriosis is a disease defined by the presence of endometrial-like tissue outside of the uterus [1]. Endometriosis is estimated to occur in up to 10% of women and those assigned female at birth during their reproductive years [2,3], with 11% of women being diagnosed by age 44 in Australia [4]. Endometriosis-related chronic pelvic pain (CPP) refers to a variety of pain symptoms including dysmenorrhea (period pain), dyspareunia (pain during sexual intercourse), fatigue, dyschezia (pain on bowel motions) and dysuria (pain on urination) [5,6,7]. People with endometriosis are often diagnosed with various comorbidities including irritable bowel syndrome, rheumatoid arthritis, psoriasis, anxiety, depression [8] and chronic fatigue syndrome [9]. The debilitating symptoms of endometriosis impact social activities, work and career progression, finances, academic studies, mental health, emotional health and sexual/romantic relationships, ultimately impacting quality of life [5,10,11,12,13]. Furthermore, the economic burden of endometriosis impacts the Australian economy, costing AUD 30,000 per woman per year, with the most predominant factor being lost productivity, directly correlated with pain severity. Internationally, this varies depending on different national economies, from USD 1459 to USD 20,239 per year.

One of the known mechanisms through which endometriosis symptoms occur is a result of repetitive deposition of endometrial-like tissue and active breakdown of endometriotic lesions, which in turn leads to an inflammatory cascade alongside the development of adhesions and scar tissue, as well as other factors, causing pelvic pain [1,14]. The inflammation-mediated pain has been correlated with high levels of cyclooxygenase-2 (Cox-2) and tumour necrosis factor alpha (TNF-α) in spinal cords and brains, alongside peripheral macrophages in a murine model [15].

Endometriosis is often characterized by the altered efficiency of progesterone and estrogen hormones, leading to progesterone resistance, as observed in people with endometriosis not responding to the use of progestins, and excess estrogen levels. This depicts an imbalance that initiates local infiltration of immune cells and inflammation [16,17]. Newly established cells resulting from inflammation activate various pathways of cell proliferation, angiogenesis, metastasis and invasion. As estrogen and progesterone receptors are responsible for proliferation and differentiation in the endometrium, imbalances result in changes in the expression of estrogen-metabolising enzymes, promoting progesterone resistance in endometriotic lesions [18].

Current treatments for endometriosis include surgical and/or medical management. Common treatments include analgesics (both opioid and non-opioid), hormonal treatments and anti-neuropathics. Hormonal medications include progestins, combined oral contraceptives, gonadotropin-releasing hormone (GnRH) agonists [19] or antagonists [20] and androgen analogues [21]. These modify the endocrine environment in both eutopic endometrium and ectopic lesions and hinder the inflammatory pathway involved in pelvic pain [21]. Commonly used anti-neuropathics include amitriptyline, pregabalin and gabapentin [22], the use of which is common among those experiencing chronic pelvic pain. Previous clinical trials [22] have shown the effectiveness of gabapentin on CPP, but currently, its effectiveness in endometriosis may not be effective due to the fact that endometriosis-associated pain tends not to be neuropathic [21]. There is currently limited evidence for the use of anti-neuropathics for endometriosis specifically; the use of anti-neuropathic medication is not recommended for the endometriosis cohort as endometriosis-associated pain should not be treated as neuropathic pain [21].

Overall, medical treatments are generally considered suboptimal by those with the disease [23,24] with concerns about the lack of effectiveness and problematic side effects of many medications for pelvic pain [25]. Opioid analgesics are not recommended for CPP due to both a lack of efficacy and safety concerns with respect to ongoing use [26]. However, despite this, they continue to be prescribed; people with endometriosis have a four times greater risk of chronic opioid use compared to those without [27], and opioids are often prescribed alongside benzodiazepines [28]. Both opioids [29] and benzodiazepines [30] present a significant risk of cognitive impairment, addiction and severe withdrawal symptoms, with a combination of these drugs significantly increasing the risk of overdose [31]. Surgery is considered a viable and common effective treatment [32] but often has significant costs, long waiting times [33] and substantial recurrence rates, even with expert endometriosis surgeons [34]. Access to surgery is even more reduced in developing countries and remote and rural locations [35].

Due to these issues, novel pain management options are considered an urgent research and clinical priority in endometriosis [36,37,38]. Limited access to surgery often leads to people with endometriosis employing self-management strategies. The use of cannabis, either illicitly or legally, is becoming a relatively popular self-management strategy in those with endometriosis, with substantial self-report data on the reduction in symptoms [39]. This review explores the potential mechanisms of action by which cannabis may modulate endometriosis symptoms, in the hope of exploring a new and effective therapeutic avenue.

The growing interest in the gut microbiota and its influence on various metabolic and inflammatory diseases poses the question of gut microbiota involvement in the modulation and/or pathogenesis of endometriosis. Studies have also reported interactions between gut microbes and the endocannabinoid system (ECS). Both of these aspects will be explored in this review.

### 1.1. Endometriosis and the Endocannabinoid System

Research into the *Cannabis* genus during the 1990s contributed to the scientific discovery of the ECS [40]. The ECS is a complex signalling system that comprises three major components: G-protein-coupled cannabinoid receptors (CB_1_, CB_2_, endocannabinoids (endogenously produced cannabinoids), including anandamide (AEA) and 2-arachidonoyl glycerol (2-AG)), ion channel transient receptor potential vanilloid 1 (TRPV1) [41] and the enzymes involved in the synthesis and catabolism of endocannabinoids [42,43,44]. Table 1 summarises the key endocannabinoids to be discussed in this paper.

Research to date has demonstrated that the ECS is involved in homeostasis and regulation via neuromodulatory activity, physiological processes such as digestion [46], immune function [47], nociception [48], appetite regulation [49], cardiovascular and respiratory function [50] and sleep–wake cycles [51].

**Figure 1:**
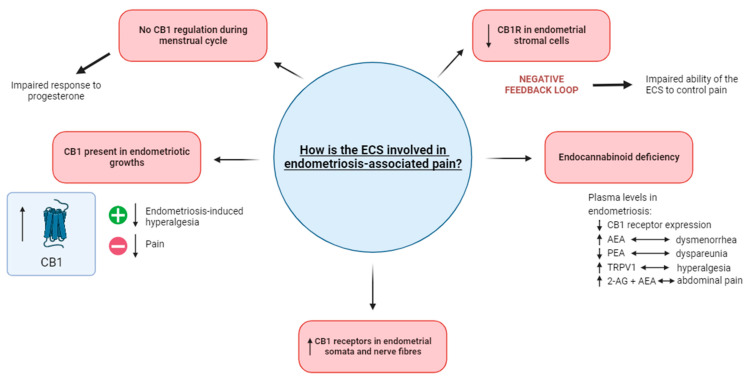
The involvement of the ECS in endometriosis-associated pain. The impaired response to progesterone in endometriosis potentially occurs as a result of CB_1_ regulation not occurring during menstruation. When present in endometriotic growths, the activation of CB1 decreases endometriosis-induced hyperalgesia while its blockage results in decreased pain. Increased levels of CB_1_ receptors have been noted in the endometrial somata and nerve fibres. An endocannabinoid deficiency in endometriosis is demonstrated in plasma levels, where there is an increased level of TRPV1 (linked with hyperalgesia) and 2-AG and AEA (linked with abdominal pain). In contrast, decreased levels of CB_1_ receptor expression and PEA (linked with dyspareunia) are also recognized as the defining features of an endocannabinoid deficiency. The decreased level of CB_1_ receptors in endometrial stromal cells is suggested to cause a negative feedback loop, possibly impairing the ability of the ECS to control pain. Abbreviations: ECS = endocannabinoid system; CB_1_ = cannabinoid receptor 1; 2-AG = 2-arachidonoyl glycerol; AEA = anandamide; PEA = palmitoylethanolamide.

The ECS influences pain modulation, making it a potential target for the treatment of chronic pain conditions such as endometriosis (Figure 1). Activation of CB_1_ and CB_2_ receptors by endocannabinoids or exogenous cannabinoids can suppress nociceptive processing and induce analgesia [52,53,54,55]. Similar observations were made in preclinical studies showing the analgesic effects of cannabinoid agonists on neuropathic pain [56,57,58]. However, clinical trials have presented mixed results in terms of the efficacy of cannabinoids in pain modulation. In a prospective randomized placebo-controlled trial, smoked cannabis (3.56% delta-9-tetrahydrocannabinol (THC)—participants smoked three cigarettes daily over a 4-day period) reduced daily pain experienced by adults with HIV-associated sensory neuropathy [59]. In contrast, the administration of Sativex, a low-dose THC (2.7 mg/100 μL) and cannabidiol (CBD) (2.5 mg/100 μL) combination oro-mucosal spray (used over an 8-week study period—drugs were titrated per patient) in adults with multiple sclerosis showed non-significant differences between the treatment and placebo groups [60]. As studies often employ different modes of administration, which affects bioavailability, the resulting effect of cannabis varies, and therefore, further studies will be required to fully understand the mechanisms and therapeutic potential of the ECS.

Endocannabinoid receptors have been found throughout female reproductive tissue [44], and the use of exogenous cannabinoids may alleviate endometriosis-associated pain [61,62]. The ECS also regulates endometriosis mechanisms such as inflammation, angiogenesis, apoptosis, endometrial hyperproliferation and fibrosis [44,61,62,63,64,65,66,67], and this may be a potential pathway by which endometriosis lesions re-occur post-surgery.

Cannabinoid receptors, specifically CB_1_ receptors, are expressed on both the somata and fibres of sensory and sympathetic neurons that innervate endometriotic growths [68]. Activation of CB_1_ receptors has been linked to a reduction in endometriosis-induced hyperalgesia, while blocking CB_1_ receptors increases pain [69]. Moreover, elevated levels of endocannabinoids, such as AEA and 2-AG, have been observed in people with endometriosis [44]. However, the expression of CB_1_ receptors in endometrial stromal cells is lower in endometriosis compared to healthy controls, suggesting a negative feedback loop that may impair the ability of endocannabinoids to control pain [70]. The levels of endocannabinoids in people with endometriosis vary across the menstrual cycle, and this is thought to be influenced by the on-demand synthesis and degradation of enzymes [71]. In the secretory phase, however, CB_1_ regulation is absent, reflecting an impaired response to progesterone levels [72], highlighting the complex nature of this disease. Table 2 outlines the findings of these key studies.

#### 1.1.1. Endocannabinoid Deficiency in Endometriosis

Endometriosis has been described by some researchers as an “endocannabinoid deficiency” [72]. Plasma levels of endocannabinoid ligands AEA, 2-AG, *N*-oleoylethanolamine (OEA) and *N*-palmitoylethanolamine (PEA) fluctuate in people with endometriosis [72]. When associating these endocannabinoids with endometriosis-associated pain severity, a correlation was noted between increased endocannabinoid ligands in plasma and decreased local CB_1_ receptor expression in people with endometriosis [72]. These levels were studied in association with common endometriosis symptoms, where it was identified that elevated levels of AEA were linked with moderate-to-severe dysmenorrhea while elevated levels of PEA were found in people with moderate-to-severe dyspareunia [72], demonstrating an interesting contrast that requires further insight. This indicates a potential dysregulation in the ECS, suggesting an altered signalling process in response to endometriosis-related pain.

#### 1.1.2. Potential Interplay of the ECS and Endometriosis

The peritoneal microenvironment is often studied in light of the establishment and progression of endometriosis. Inflammatory cytokines (interleukin-1β (IL-1β), interleukin-6 (IL-6), insulin-like growth factor (IGF-1) and tumour necrosis factor-α (TNF-α) are observed to be at a higher concentration in the peritoneal fluid of people with endometriosis [43,73,74,75]. The production of proinflammatory cytokines may occur as a result of disease-modified macrophages producing IGF-1, as high concentrations of IGF-1 were found in the peritoneal fluid of people with endometriosis [15]. This in turn leads to people experiencing hyperalgesia, which has been correlated with altered expression of TRPV1 in the peritoneum of people with endometriosis [76]. Levels of 2-AG and AEA have also been observed to be significantly higher in the peritoneal fluid of people with endometriosis, and this was associated with relatively higher abdominal pain [77]. The protective role of the ECS was observed in people with endometriosis, where immunohistochemistry revealed that cannabinoid agonists inhibit endometrial cell proliferation [65]. In contrast, previous studies have also shown the involvement of the ECS in endometriosis modulation and progression (Figure 1). ECS modulation in the innervation of ectopic uterine growths was demonstrated through the abundance of CB_1_ receptors on sensory and sympathetic fibres innervating ectopic growths, dorsal root ganglia and coeliac ganglia [44]. This was also observed in the epithelial cells in ovarian endometriotic lesions [78]. Such studies demonstrate the complex and contradictory role of the ECS in pain modulation, warranting more research in this area to understand the mechanisms behind the interaction of the ECS and endometriosis.

#### 1.1.3. Exogenous Cannabinoids

The intricate and multifaceted role of the ECS in pain modulation has spurred investigations into the potential impact of exogenous cannabinoids on pain management. There is currently only one trial on medicinal cannabis for endometriosis, where a CBD isolate oil (100 mg/mL) is being compared to CBD and vaporized THC cannabis flower (ACTRN12622001560785). This is a randomized, controlled feasibility study, assessing the usefulness of medicinal cannabis on people with endometriosis, who present to the emergency department. While clinical trials are yet to be conducted, preclinical studies have explored the effects of the exogenous cannabinoid delta-9-tetrahydrocannabinol (THC) on pain management. In murine models of endometriosis, repetitive administration of botanically derived THC demonstrated protective properties [79]. It hindered the growth of ectopic endometrial tissue while alleviating mechanical hypersensitivity in the caudal abdominal region. In an experimental mouse model, varying concentrations of cannabidiol (CBD) were found to significantly reduce endometriotic implant surface area, alongside proinflammatory cytokine levels, including IL-6 and TNF-α [80]. A similar study showed that CBD administration reduced endometriotic lesion diameter, volume and area in vivo, demonstrating antioxidant effects by reducing lipid peroxidation [81]. These promising findings highlight the potential therapeutic benefits of CBD and THC for endometriosis-associated pain, warranting the need for human studies.

**Table 2:** A summary of recent studies on endometriosis and the endocannabinoid system.

Author	Year	Type of Study	Endocannabinoid/Cannabinoid	Methodology	Findings
Andrieu et al. [77]	2022	Human—endometriosis	2-AG and AEA	LCMS analysis of endocannabinoids ELISA analysis of cytokines	Increased abdominal pain associated with a high level of 2-AG and a low level of AEA in the peritoneal fluid of people with endometriosis.
Sanchez et al. [72]	2016	Human—endometriosis	CB_1_	PCR analysis of menstrual cups of people with endometriosis	The absence of CB_1_ regulation in endometriosis in the secretory phase might reflect an impaired response to progesterone.
Rocha et al. [76]	2011	Human—endometriosis	TRPV1	Immunohistochemistry analysis of rectouterine peritoneum	Correlation identified between greater expression of TRPV1 in the peritoneum of people with endometriosis who experience CPP compared to those who do not.
Bilgic et al. [65]	2017	Human endometrial archive samples	Cannabinoid agonists	Immunohistochemistry analysis	Cannabinoid agonists inhibit endometriotic cell proliferation, modulating apoptosis of endometriotic cells.
Okten et al. [80]	2023	Female Wistar albino female rats	CBD	Immunohistochemical staining	CBD reduced endometriotic implant surface area and proinflammatory cytokine levels.
Escudero-Lara et al. [79]	2020	Female C57Bl/6J mice	THC	Immunostaining	Repetitive administration of THC hindered ectopic endometriotic growths, alleviating hypersensitivity.
Genovese et al. [81]	2022	Sprague Dawley rats	CBD	Histological analysis ELISA	CBD reduced endometriotic lesion diameter and demonstrated antioxidant effects, as viewed by downregulated expression of MMP-9, iNOS and TGFβ.
ACTRN12622001560785	2023	Human	THC + CBD	Various questionnaires being used	Medicinal cannabis and endometriosis trial registration.

Abbreviations: 2-AG = 2-arachidonoyl glycerol; AEA = anandamide; CB_1_ = cannabinoid receptor 1; TRPV1 = transient receptor vanilloid 1; chronic pelvic pain (CPP); CBD = cannabidiol; THC = delta-9-tetrahydrocannabinol; LCMS = liquid chromatography-mass chromatography; ELISA = enzyme-linked immunosorbent assay; PCR = polymerase chain reaction; MMP-9 = matrix metallopeptidase-9; iNOS = inducible nitric oxide synthase; TGFβ = transforming growth factor beta.

### 1.2. The Endocannabinoid System and the Gut Microbiota

The gut microbiota refers to the collection of trillions of microorganisms, including bacteria, archaea, viruses and fungi. The gut microbiota plays a crucial role in human health and disease, impacting immunomodulation and inflammatory processes [82].

In humans, the gut microbiota is dominated by four bacterial phyla: *Firmicutes*, *Bacteriodetes*, *Actinobacteria* and *Proteobacteria* [83]. As the phylotypic composition of these can vary amongst individuals [84], it can be implied that each host has a unique biological relationship with its gut microbiota, thereby influencing the risk of disease [85,86]. The composition of the gut microbiota in physiological processes also changes with age, implicating long-term health outcomes.

Given the nascent nature of this field of research, there is currently a scarcity of studies examining the relationship between the ECS, the gut microbiota and endometriosis. However, studies have shown the involvement of the ECS in the gut microbiota in metabolic and inflammatory disorders in a bidirectional manner.

The gut is part of the ECS. CB_1_ receptors have been localised in the gut epithelium, smooth muscle, submucosal myenteric plexus and myenteric ganglia [87,88]. CB_2_ receptors have been detected in the plasma cells and macrophages of the gastrointestinal (GI) mucosa and submucosa, as well as intestinal epithelial cells in the GI mucosa [87,89]. While CB_1_ receptor activation is associated with appetite regulation and relief from nausea and vomiting, CB_2_ receptor activation mediates inflammation [90]. Endocannabinoids are known to be synthesised in various parts of the gut and their levels fluctuate, based on metabolic and inflammatory status. Endocannabinoids and exogenous cannabinoids exert opposite effects on gut permeability. For instance, when examining decreased permeability as a result of inflammation, it was demonstrated that 2-AG and AEA increased permeability, while THC and CBD decreased permeability [91,92]. 

#### 1.2.1. Interactions between Gut Microbial Communities and Endocannabinoids

While the interactions between gut microbiota and endocannabinoids have mainly been investigated in preclinical models, studies have linked these interactions to beneficial effects in disease states such as inflammatory bowel disorder (IBD). The administration of *Lactobacillus acidophilus* in mice led to an increase in CB_2_ expression in intestinal epithelial cells, resulting in analgesic effects, thus decreasing visceral pain [89]. Furthermore, the administration of a probiotic mixture containing *Bifidobacteria*, *Lactobacilli* spp. and *Streptococcus thermophilus* in zebrafish resulted in an upregulation of CB_1_ and CB_2_ expression, which then led to anti-inflammatory effects [93,94].

*Akkermansia muciniphila* is a Gram-negative anaerobic mucus-degrading bacterium, abundantly found in healthy intestinal mucosa. *A. muciniphila* has been found to modulate gut barrier integrity. Its protective effects have been cited in relation to the ECS. A study administering *Akkermansia muciniphila* in mice fed a high-fat diet led to an increase in 2-AG, 2-OG and 2-PG levels [95]. *A. muciniphila* has been shown to regulate CB_1_ mRNA in Caco-2 cells through increased production in outer membrane vesicles, which prevent the development of metabolic disorders such as obesity [96]. Moreover, a decrease in CB_1_ activity resulted in reduced circulating lipopolysaccharide (LPS) levels, thus improving the inflammatory cytokine profile and intestinal permeability. This occurred alongside enhanced *A. muciniphila* and *Lachnospiraceae* levels in the gut. The gut microbiota profile of obesity is characterised by a *Firmicutes:Bacteriodetes* ratio, whereby there is an increased abundance of *Firmicutes* and a reduced abundance of *Bacteriodetes*. When chronically treating obesogenic mice with THC, this ratio was shifted, and an increase in the abundance of *A. muciniphila* was observed, demonstrating the protective effects of exogenous cannabinoids [97]. Further investigation is required to understand how *A. muciniphila* interacts with endocannabinoids and the outcomes of this on inflammation-associated disorders.

Studies have also examined the relationship between the ECS and gut microbial metabolites. For instance, endocannabinoids have been found to mediate the anti-inflammatory effects of SCFAs. This association was observed in an exercise intervention [98] where an increase in SCFAs (including butyrate) and SCFA-producing bacteria (such as *Bifidobacterium*) was correlated with a decrease in proinflammatory cytokines TNF-α and IL-6. Furthermore, an increased abundance of SCFA-producing bacteria, such as *Bifidobacterium*, was positively associated with increases in endocannabinoids AEA, PEA and OEA, which were correlated with CB_1_ receptor levels. Table 3 illustrates the imbalances of SCFAs currently reported in endometriotic faeces. These studies provided an indication of how the association between the ECS and the gut microbiota can be potentially utilised to improve gut inflammation. The recent literature on the link between the ECS and gut microbiota is summarised in Table 4.

#### 1.2.2. The ECS–Gut–Brain Axis

As CB_1_ receptors are found in the CNS, the ECS is involved in signalling, modulating various physiologic and homeostatic processes. While the ECS–gut–brain axis has not been studied extensively, recent studies have considered the involvement of the ECS–gut–brain axis in exercise interventions, processes of food intake and disease states such as Alzheimer’s disease.

While investigating the microbiota-dependent gut–brain pathway on exercise, it was shown that the CB_1_-expressing TRPV1 sensory neurons are triggered by fatty acid amide metabolites from gut bacteria such as *Lachnospiraceae* and *Eubacterium* [99]. The protective effects of *Lachnospiraceae* have been well-studied in various diseases, including ulcerative colitis [100,101]. This led to analgesic effects observed after exercise, demonstrating the interaction of intestinal microbial colonization and peripheral CB_1_ signalling.

Endocannabinoids such as AEA and CB_2_ receptors and enzymes such as fatty acid amide hydrolase (FAAH) influence retrograde signalling (observed in Alzheimer’s disease) in the brain. Here, inhibitory feedback regulates neurotransmitter release [45]. Moreover, as AEA induces gut permeability, its overexpression is thought to cause a “leaky” gut, resulting in metabolic endotoxemia [102]. As this condition leads to the release of toxins such as LPS which may cross the epithelial barrier, an inflammatory signalling pathway may develop, impacting the CNS and resulting in neuroinflammation [103].

CB_1_ binding leads to increased food intake. CB_1_ receptors are present in cells of the lining of the intestinal epithelium. When CB_1_ receptor activity is heightened, in the small-intestinal epithelium, the release of cholecystokinin-8 (CCK-8) is inhibited, resulting in delayed satiation and overeating (in diet-induced obesity) [104], highlighting the indirect control mechanism of endocannabinoids in gut–brain neurotransmission.

#### 1.2.3. The ECS in IBD

The use of cannabis for abdominal pain relief and other IBD symptoms is prevalent, with studies reporting use amongst 70–95% of people with IBD [90,105,106,107]. However, several studies have reported similar results when using endocannabinoids and/or exogenous cannabinoids in therapies for IBD. In an analysis of biopsies of paediatric patients, AEA levels were found to be significantly decreased in inflamed IBD mucosa [108]. A significant increase in the expression of CB_2_ receptors has also been observed in the biopsies of Crohn’s disease ileum and in rectum biopsies of ulcerative colitis, colocalised with T-lymphocyte infiltration [109]. Similarly, murine IBD studies demonstrated that increasing the availability of the endogenous CB_1_ and CB_2_ receptor agonists diminishes visceral pain [110,111,112]. Nonetheless, the issue remains that such implications have not translated to clinical trials, as noted in one study where people with IBD using cannabis reported higher incidences of abdominal pain and arthralgias [113]. Further research is required to develop insights into what may be causing these negative side effects and utilisation of the ECS as a therapy for IBD.

There is a high prevalence of inflammatory bowel syndrome (IBS) and its associated symptoms in people with endometriosis; in one study, 52% (194/373) of those with endometriosis had diagnosed IBS [114], where those experiencing minimal–mild endometriosis often report more severe IBS symptoms, as compared to those with moderate–severe endometriosis. Similarly, of 160 people with diagnosed IBS, 59 had a history or recent diagnosis of endometriosis [115]. People with endometriosis are reported to have a threefold increase in the likelihood of developing IBS [116]. Such studies demonstrate the importance of understanding the mechanisms through which inflammatory bowel diseases and endometriosis interact and thus the need for treatments targeting both diseases [116].

**Table 4:** A summary of recent literature on endocannabinoids and gut microbes.

	Year	Type of Study	Endocannabinoid/Cannabinoid + Microbiota	Methodology	Findings
Strisciuglio et al. [109]	2023	Human—Crohn’s disease	CB_2_ receptors	Western blot immunofluorescence	Increased expression of CB_2_ receptors in ileum of people with Crohn’s disease.
Vijay et al. [98]	2021	People with knee osteoarthritis	2-AG, OEA, AEA, PEA *Bifidobacterium*, *Coprococcus*, *Faecalibacterium*, *Colinsella*	Metabolomic analysis Gut microbiome analysis Gene expression assay	An association between increased levels of SCFAs with circulating levels of endocannabinoids, higher microbiome diversity and low levels of proinflammatory *Colinsella.*
Pagano et al. [117]	2019	Pediatric patients with ulcerative colitis and male adult CD1 mice	Cannabidivarin TRPA1	RT-PCR	In a TRPA1 antagonist manner, cannabidivarin regulates systemic inflammation and intestinal permeability.
Di Sabatino et al. [108]	2011	Human—Crohn’s disease Mucosal samples	AEA	HPLC-MS Wound healing scratch assay Immunohistochemistry	Significantly low levels of AEA in inflamed gut mucosa.
Grill et al. [88]	2019	C57BL/6 mice	CB_1_ receptors	In situ hybridisation Immunohistochemistry	Changes in gene expression of CB_1_ and CB_2_ receptors, GPR-55 and monoglycerol lipase in the gut after LPS treatment, demonstrating involvement in intestinal and systemic inflammation. These were observed in comparison to CB_1_ and MGL knockout mice. High expression of CB_1_ receptors in the submucosal and myenteric plexus. Reduced MGL expression in the ileum following LPS treatment. GPR-55 mRNA present alongside T-cell and macrophage markers in the ileum of healthy and treated mice.
Argueta and DiPatrizio [104]	2017	Male C57BL/6Tac mice	CB_1_ receptor, 2-AG and AEA	LCMSGene expression analysis	In diet-induced obesity, the increase in CB_1_ receptor activity inhibits CCK-8, resulting in delayed satiation and overeating.
Mehrpouya-Bahrami et al. [118]	2017	Male C57BL/6J mice	CB_1_ receptor *Akkermansia muciniphila*, *Lachnospiraceae*, *Erysipelotrichaceae*,	16s RNA metagenomics	Blocking CB_1_ receptor activity resulted in decreased LPS activity, enhancing anti-inflammatory effects by increasing the abundance of *A. muciniphia, Lachnospiraceae* and *Erysipelotrichaceae*.
Cluny et al. [119]	2015	Male C57BL/6N mice	*Firmicutes,* *Bacteriodetes* *A. muciniphila* THC	qPCR	Chronic administration of THC in obesogenic mice increased the *Firmicutes:Bacteriodetes* ratio and the abundance of *A. muciniphila.*
Sakin et al. [112]	2015	Adult male Balb-C mice and Sprague Dawley rats	CB_1_ and CB_2_ receptors	Colorectal distension test Nociceptive testing	Availability of CB_1_ and CB_2_ receptors diminishes visceral pain.
Kiran, Rakib, Moore and Singh [120]	2022	Female C57BL/6 mice	CB_2_ inverse agonist SMM-189	Flow cytometry analysis Western blot analysis Histology	CB_2_ inverse agonist SMM-189 suppressed colitis, while ameliorating the loss of body weight, reducing the inflammatory disease score and disease severity.
Dohnalova et al. [99].	2022	C57BL/6J mice	CB_1_	Fibre photometry analysis DRG extraction, culture and calcium imaging Amplex fluorometry analysis PCR, qPCR, RNA-seqTranscriptional profiling	Fatty acid amide metabolites trigger CB_1_-expressing TRPV1 sensory neurons, thus elevating dopamine levels during exercise.
Jamontt, Molleman, Pertwee and Parsons [121]	2010	Male Charles River Wister rats Distal colon tissue	CBD + THC	In vitro evaluation MPO assay BCA protein assay	CBD and THC reduced inflammation and functional disturbances by reducing the release of TNFa, IFNγ and nitric oxide in vitro and in vivo.
Borelli et al. [122]	2009	Male ICR mice	CBD	Western blot ELISA LCMS	CBD reduced colon injury and decreased expression of inflammatory markers, including nitric oxide synthase and reactive oxygen species.
Alhamoruni et al. [92]	2012	Caco-2 cells	CB_1_ and CB_2_ receptors, TRPV1, PPARγ and PPARα THC and CBD	Measurements of transepithelial electrical resistance	THC and CBD accelerated recovery of cytokine-induced intestinal permeability.
Distrutti et al. [94]	2014	Zebrafish	*Bifidobacteria, Lactobacilli* spp. and *Streptococcus thermophilus*	TUNEL assay	Administration of a probiotic mixture containing *Bifidobacteria, Lactobacilli* spp. and *Streptococcus thermophilus* in zebrafish led to an increase in CB_1_ and CB_2_ expression.
Gioacchini et al. [93]	2017	Adult male zebrafish	*Bifidobacteria, Lactobacilli* spp. and *S. thermophilus* *Bacteriodetes* and *Actinobacteria*	RT-PCR Immunohistochemistry	Administration of a probiotic mixture containing *Bifidobacteria, Lactobacilli* spp. and *S. thermophilus* in aged zebrafish resulted in an increased abundance of *Bacteriodetes* and *Actinobacteria*, alongside increases in CB_1_, demonstrating anti-inflammatory effects.

Abbreviations: CB_2_ = cannabinoid receptor 2; 2-AG = 2-arachidonoyl glycerol; OEA = *N*-oleoylethanolamine; AEA = anandamide; PEA = *N*-palmitoylethanolamine; TRPA-1 = transient receptor potential ankyrin type 1; CB_1_ = cannabinoid receptor 1; THC = delta-9-tetrahydrocannabinol; PPARγ = peroxisome proliferator-activated receptor gamma; PPARα = peroxisome proliferator-activated receptor alpha; CBD = cannabidiol; RNA = ribonucleic acid; HPLC-MS = high protein liquid chromatography—mass spectrometry; RT-PCR = reverse transcriptase polymerase chain reaction; PCR = polymerase chain reaction; qPCR = quantitative polymerase chain reaction; LCMS = liquid chromatography mass spectrometry; TUNEL = terminal deoxylnucleotidyl transferase dUTP nick end labelling; DRG = dorsal root ganglia; RNA-seq = RNA sequencing; SCFA = short-chain fatty acid; GPR55 = G-couple protein receptor 55; MGL = monoacyl glycerol lipase (MGL); CCK-8 = cholecystokinin-8; LPS = lipopolysaccharide; IFNγ = interferon gamma; TRPV-1 = transient receptor vanilloid 1; TNF-α = tumour necrosis factor alph.

### 1.3. Endometriosis and the Gut Microbiota

**Figure 2:**
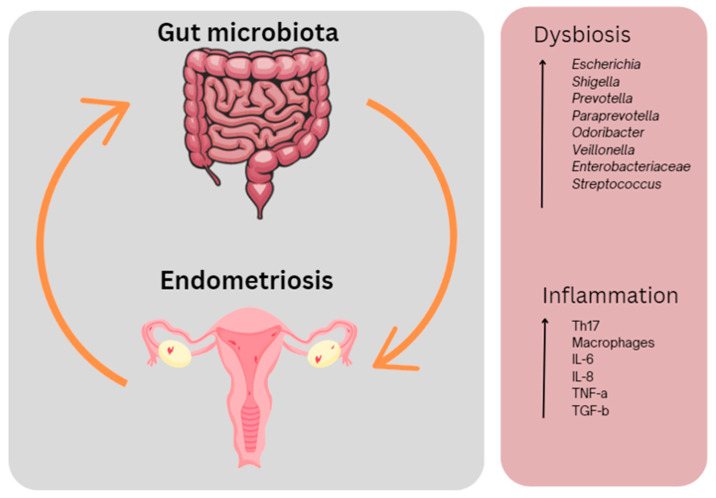
The interplay between the gut microbiota and endometriosis. Dysbiosis in the gut and inflammation occurring both in the gut and in the peritoneal cavity results in endometriosis-associated symptoms including pain and fatigue. This figure was created using Canva.com (accessed on 27 September 2023).

A bidirectional relationship between endometriosis and gut microbiota has been proposed (Figure 2). The current literature on the potential role of gut microbiota in endometriosis is summarised in Table 5. As gut microbes and their metabolites are involved in various immune, metabolic and epithelial functions, imbalances in the gut microbiota can trigger an inflammatory response through specific inflammatory immune cell recruitment, proinflammatory cytokine production and compromised immune surveillance. These processes may be involved in some of the changes in inflammatory markers seen in endometriosis, including raised levels of IL-6 and dysfunction of macrophages [123,124,125,126].

#### 1.3.1. Microbial Dysbiosis in Endometriosis

**Figure 3:**
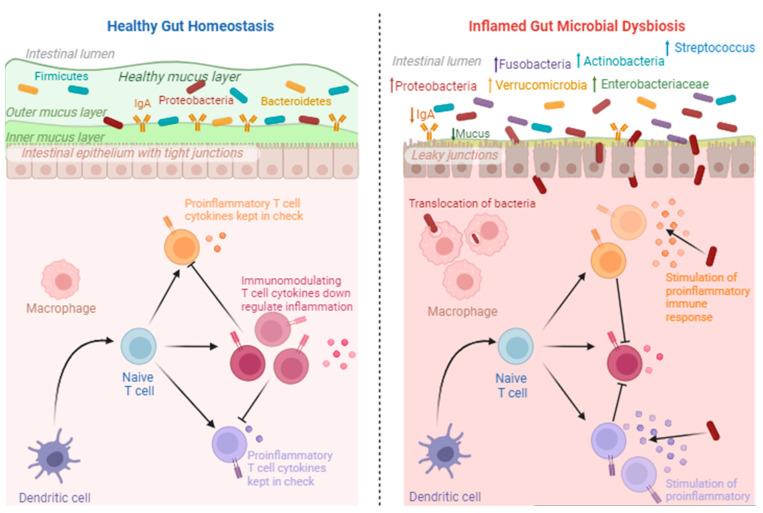
Healthy gut homeostasis vs. inflamed gut microbial dysbiosis. The downregulation of inflammatory cytokines occurs under healthy conditions. However, under inflammatory conditions, proinflammatory cytokines are upregulated as a result of increases in pathogenic microorganisms. This image was created using Biorender.com (accessed 27 September 2023).

In the endometriotic state, larger percentages of bacterial phyla *Proteobacteria*, *Verrucomicrobia*, *Streptococcus* or *Fusobacteria* have been reported (Figure 3). Increases in *Enterobacteriaceae, Streptococcus* and *E. coli* have been identified as dominating phyla in endometriosis cohorts [127]. Recently, *Fusobacterium* has been suggested to contribute to the pathogenesis of endometriosis [128]. The dominance of *Shigella* has also been noted [129]. Such microbial communities are known to be involved in the degradation of estrogen by producing β-glucuronidase and β-glucosidase [130], inevitably resulting in the development of a high-estrogen environment, promoting the progression of endometriosis [131,132].

An increase in *E. coli* is attributed as a biomarker of endometriosis, and this has led to the “bacterial contamination” theory. High levels of *E. coli* have been found in faecal [133] and menstrual blood samples [134] of participants with endometriosis. *E. coli* is known to trigger TLR4-mediated growth and progression of endometriosis, resulting in pelvic inflammation [135]. This occurs through the production of LPS, triggering secretion of secondary inflammatory mediators such as NF-κB, in the peritoneal cavity, therefore, resulting in the development and progression of endometriosis.

Further research is required to unravel the intricate dynamics between the microbiota and its influence on endometriosis progression. Such investigations would not only grow the understanding of the way in which the microbiota promote endometriosis pathogenesis but also the development of targeted therapies to improve the quality of life of those with endometriosis.

#### 1.3.2. Microbial Dysbiosis and Endometriosis Symptoms

Research has demonstrated correlations with specific microbes and bacterial phyla impacting symptoms in those with endometriosis. Previous studies have shown the estrogen–gut–brain axis, which is thought to influence the development of chronic stress in people with endometriosis via the activation of β-adrenergic signalling [136]. This has been associated with dysbiosis on a genus level, particularly a decrease in *Paraprevotella*, *Odoribacter*, *Veillonella* and *Ruminococcus* [136], which are often viewed as biomarkers of endometriosis [137]. A murine study showed that a decrease in *Ruminococcus* is negatively correlated with apoptosis of endometriotic epithelial cells and increased IL-6 levels, resulting in peritoneal inflammation [138]. *Prevotella* has been found in high abundance in people with endometriosis, especially in those experiencing gastrointestinal symptoms, and is associated with constipation, bloating, flatulence, vomiting and nausea [139]. As many studies report a correlation between gut microbiota imbalances and endometriosis, continued and extensive research is essential in understanding the full potential of harnessing the gut microbiota for the treatment of endometriosis and its associated symptoms.

The vaginal microbiota is dominated by healthy *Lactobacillus*, which maintains an acidic and protective environment, preventing the growth of pathogenic bacteria. However, in endometriosis, the protective environment maintained by vaginal microbiota is impacted by an abundance of *Gardnerella, Escherichia*, *Shigella*, *Ureoplasma* [129], *Streptococcus*, *Moraxellae*, *Staphilococcus* and *Enterobacteria*, coupled with a lowered abundance of *Lactobacillus* [140]. These imbalances have been correlated with endometriosis-associated pelvic pain [141,142]. An understanding of the vaginal microbiota in endometriosis, in correlation with the ECS, is required for the characterisation of treatments that may be prescribed for people with endometriosis.

**Table 5:** A summary of recent literature on the gut microbiota and people with endometriosis.

	Year	Microbiota	Methodology	Findings
Svensson et al. [139]	2021	*Prevotella*, *Bacilli*, *Bacteriodia*, *Clostridia*, *Coriobacteria* and *Gammaproteobacter*	16s rRNA sequencing	*Prevotella* has been associated with constipation, flatulence, bloating, vomiting and nausea in endometriosis. High abundance of *Lactococcus* (*Bacilli)*, lower abundance of *Odoribacter* and higher abundance of *Prevotella* in endometriosis.
Sandstrom et al. [138]	2020	*Rumincoccus*	16s rRNA sequencing	Decrease in *Ruminococcus* correlated with an increase in IL-6 in a murine model of endometriosis, resulting in peritoneal inflammation.
Ata et al. [129]	2019	Complete absence of *Atopobium*, *Gardnerella*, *Streptococcus*, *Escherichia*, *Shigella*, *Ureoplasma*	PCR amplification 16s rRNA sequencing	Absence of *Atopobium* in vaginal and cervical microbiota. Increased *Gardnerella* in cervical microbiota. Dominant gut microbiota in endometriosis group—*Escherichia* and *Shigella*. Predominant population of lower genital tract—*Lactobacillus*. *Alloprevotella* significantly decreased in the cervix.
Xu et al. [136]	2017	*Paraprevotella*, *Odoribacter*, *Veillonella* and *Ruminococcus*	16s rRNA sequencing Immunohistochemistry	The development of chronic stress in people with endometriosis occurs through the activation of β-adrenergic signalling, which occurs as a result of dysbiosis—decrease in specific unknown genus.
Khan et al. [134]	2010	*E. coli*	ELISA of macrophages from peritoneal fluid and epithelial/stromal cells from biopsy specimens of eutopic/ectopic endometria of women with and without endometriosis RT-PCR	Menstrual blood of people with endometriosis has a higher concentration of *E. coli* compared to healthy controls. An infiltration of macrophages in eutopic/ectopic endometria of people with endometriosis was noted.

Abbreviations: rRNA = ribosomal ribonucleic acid; ELISA = enzyme-linked immunosorbent assay; RT-PCR = reverse transcriptase polymerase chain reaction; IL-6 = interleukin-6.

## 2. Conclusions and Future Directions

This review summarises the complex relationship between endometriosis, the ECS and the gut microbiota. While studies have not completely deciphered the molecular basis of these relationships, the current literature demonstrates the vital roles that the ECS and the gut microbiota play in potential implications in preventing the development of endometriosis as well as effective therapeutic strategies. The mechanisms of the ECS in both endometriosis and the gut microbiota were explored. This study demonstrated current knowledge on how the dysregulation of the endocannabinoid system and the gut microbiota influence the progression of endometriosis. The fluctuation of endocannabinoids in plasma, correlated with endometriosis-associated pain severity, demonstrates the dysregulation of the ECS in endometriosis. This dysregulation has been linked with innervation of ectopic uterine growths. Furthermore, variable levels of endocannabinoids prevalent in female reproductive tissue ultimately result in endometriosis-related symptoms. Here, the administration of THC and CBD depicted the protective nature of exogenous cannabinoids on endometriosis. Moreover, the protective effects of the ECS on the gut were observed by increases in endocannabinoids, including 2-AG, resulting in decreased inflammation and improved gut permeability. Microbial imbalance in the gut and menstrual blood have been directly linked to bloating in endometriosis. Importantly, increases in specific bacterial phyla were associated with increases in inflammatory markers such as TNF-α and IL-6. Further research understanding the mechanisms, influence on inflammation and analysis of endocannabinoids and exogenous cannabinoids is required for the development of treatments of various diseases including endometriosis. While the impact of cannabinoids in endometriosis has been investigated, future studies are needed to comprehend how cannabinoids influence endometriosis-related pain and symptoms and how this can be implemented in the clinic, following efficacy and safety studies. This may be done through the implementation of exogenous cannabinoids, which have been shown to exert protective effects. An understanding of various inflammatory diseases was used to show the potential therapeutic role of the gut microbiota while paving new avenues for further research. While there is currently limited understanding on the way that the ECS and gut microbes interact with each other, a comprehensive understanding of this may allow both to be harnessed in exploring therapeutic avenues. Finally, the relationship between the gut microbiota and endometriosis was explored, highlighting the need for further investigations of how cannabinoids may influence gut microbiota in endometriosis.

## Figures and Tables

**Table 1:** Key endocannabinoids and related information.

Endocannabinoid	Abbreviation	Roles
Cannabinoid receptor 1	CB_1_	G-protein coupled receptor found on cells in the central and peripheral nervous system—involved in processes of mood, appetite, learning, memory and pain [45]
Cannabinoid receptor 2	CB_2_	G-protein coupled receptor found on cells in the central and peripheral nervous system—expressed during active inflammation [45]
Anandamide	AEA	Agonist towards CB_1_ and CB_2_ receptors [45]
Arachidonoyl glycerol	2-AG	Agonist towards CB_1_ and CB_2_ receptors [45]
Transient receptor potential vanilloid 1	TRPV1	Ion channel involved in endocannabinoid signalling [45]
*N*-oleoylethanolamine	OEA	Fatty acid ethanolamine—involved in food intake, inflammation and pain [45]
*N*-palmitoylethanolamine	PEA	Low affinity for CB_1_, CB_2_ and TRPV1—induces peripheral antinociception upon activating CB_1_ and CB_2_ [45]
Fatty acid amide hydrolase	FAAH	Metabolises endogenous ligands [45]
Monoglyceride lipase	MGL	Metabolises endogenous ligands [45]

**Table 3:** A comparison of SCFAs found in healthy faeces and in endometriotic faeces, showing imbalances observed in faecal samples.

SCFAs in Healthy Faeces	SCFAs in Endometriotic Faeces
↑Acetate ↑Propionate ↑Butyrate	↑Acetate ↑Propionate ↓n-butyrate ↓iso-butyrate ↓valerate

## Data Availability

Not applicable.
